# Design of Segmented Ultra-Wideband TEM Horn Antenna for Calibration of Wideband Electromagnetic Pulse Sensors

**DOI:** 10.3390/s25123599

**Published:** 2025-06-07

**Authors:** Tianchi Zhang, Yongli Wei, Yuan Wang, Changjiao Duan, Lihua Wang, Zongxiang Li, Xiao Li, Xin Li, Baofeng Cao

**Affiliations:** 1 State Key Laboratory of Chemistry for NBC Hazards Protection, Beijing 102205, China; 2 College of Information and Communication Engineering, Harbin Engineering University, Harbin 150001, China

**Keywords:** ultra-wideband antenna, segmented design, wideband electromagnetic pulse, sensor calibration

## Abstract

Wideband electromagnetic pulse detection is a crucial method for lightning disaster monitoring. However, the random nature of lightning events presents challenges in fulfilling real-time calibration requirements for electromagnetic pulse sensors. This paper introduces a segmented ultra-wideband TEM horn antenna tailored for portable calibration experiments in electromagnetic pulse detection systems. The radiating plates feature a four-section polygonal design, and an end-loaded metal plate is integrated to reduce reflection signal interference. Rigorous simulation analyses were performed on three key factors impacting antenna radiation performance: aperture impedance, tapering profile, and end loading configuration. Experimental results show that the designed antenna achieves a peak field strength of 48.9 V/m at a 10 m distance, with a rise time of 0.87 ns and a full width at half maximum of 1.75 ns. The operating frequency ranges from 48 MHz to 150 MHz, with main lobe beamwidths of 43° and 83° in the *E*-plane and *H*-plane radiation patterns, respectively. These parameters meet the technical requirements for electromagnetic pulse sensor calibration experiments.

## 1. Introduction

In the field of lightning electromagnetic pulse (LEMP) detection, wideband electromagnetic pulse sensors can accurately identify, time, and locate LEMP events. These capabilities are crucial for disaster early warning and assessment. However, the randomness and unpredictability of natural lightning events pose challenges for calibrating wideband electromagnetic pulse sensors. To perform calibration and validation tasks effectively, a wideband electromagnetic pulse simulation system with adjustable power output is required. A typical electromagnetic pulse simulator consists of a fast raise time pulse source and a pulse radiating antenna. As the core device for generating radiated fields, the pulse radiating antenna must exhibit ultra-wideband characteristics and excellent directivity to effectively simulate the time-domain and frequency-domain features of wideband electromagnetic pulses.

TEM horn antenna is a typical ultra-wideband (UWB) antenna characterized by its structure resembling gradually flaring metal plates. It offers advantages such as good directional radiation capability, high peak power capacity, and simple structure [1]. In recent years, significant progress has been made in design innovation and performance optimization of TEM horn antennas [2,3,4]. Miralles et al. [5] developed a 3D-printed TEM horn antenna that achieves ultra-wideband radiation in the 5~40 GHz frequency range, with a voltage standing wave ratio (VSWR) below −10 dB and a gain of approximately 10 dBi. Wang et al. [6] proposed an ultra-wideband axial radiation TEM horn antenna mounted on a cylindrical conductor, achieving a bandwidth ratio of 16:1 (1~16 GHz) and a gain range of 3.5~11.5 dBi. To address signal distortion during wideband radiation, Bobreshov et al. [7] utilized non-uniform dielectric material filling within the antenna, simultaneously enhancing its directivity and gain. Research on TEM horn antenna array technology has also yielded notable results. Cazden et al. [8] combined a TEM horn with a loop antenna, achieving miniaturization while maintaining good impedance matching and gain performance across 1.55~13 GHz. Kaloshin et al. [9] investigated a 2D periodic UWB TEM horn antenna array with feedlines. Under in-phase operation, the array demonstrated matching bandwidth ratios of 19:1 (*H*-plane) and 15:1 (*E*-plane), with reflection coefficients below −10 dB over the 0.35~6.6 GHz range. However, the operating frequencies of these antennas tend to be high, generally exceeding the very high frequency (VHF) range. To enable antennas to cover the VHF band, researchers have conducted many fruitful explorations [10,11,12]. Ding et al. [13] investigated a broadband VHF/UHF Double-Whip Antenna. It consists of two whip antennas, with heights of 1.6 m and 0.3 m, and operates from 30 MHz to 520 MHz with a gain range of 4.2 dBi to 6.8 dBi. Mirmozafari et al. [14] studied a Compact Wideband Multi-Beam Antenna. By combining the radiation patterns of a monopole and a loop antenna, it achieved good impedance matching and wide bandwidth between 225 MHz and 450 MHz. Gao et al. [15] explored a conformal VHF log-periodic balloon antenna. It comprises eight conformal bowtie dipole elements mounted on a cylindrical balloon (1 m in diameter and 2 m in length), covering 60~233 MHz with an average gain of approximately 4.5 dBi.

In high-power UWB pulsed radiation systems, electromagnetic pulse radiation devices based on TEM horn prototypes have emerged, including dielectric lens antennas, TEM horn arrays, and impulse radiating antennas [16,17,18]. Ostashev et al. [19] designed an antenna system comprising an array of four TEM horns. Each horn was connected to the excitation oscillator output via a matched-impedance twin-line section to ensure efficient signal transmission. By employing a non-uniform asynchronous excitation technique, the energy distribution across the aperture was optimized, enhancing energy conversion efficiency and radiation performance. Experimental results demonstrated an aperture efficiency of approximately 63%. To address the issues of large end reflection and low-frequency radiation efficiency in TEM horn antennas, Zhou Xing et al. [20] utilized time-domain analysis methods to investigate low-frequency compensation approaches involving terminal and back-loaded resistors. Structural parameters such as antenna dimensions and plate apex angles were optimized, achieving a measured peak field strength exceeding 60 kV/m. To improve the efficient potential of radiation systems, Efremov et al. [21] developed a high-power 16-element TEM horn array system. This antenna employed coaxial cable feeding and incorporated a wave impedance transformer to match the output wave impedance with the total wave impedance of the antenna feedlines, ensuring high-efficiency energy transfer. The system ultimately achieved an efficient radiation potential of 690 kV. For enhanced voltage withstand capability in compact antennas, Fedorov et al. [22] designed a 4-element TEM antenna array with dimensions of approximately 30 cm × 30 cm. The array was housed in an SF_6_ gas-filled dielectric capacitor to prevent electrical breakdown, operating across 100 MHz~6 GHz with an efficient radiation potential of 300 kV. Garnov et al. [23] created a transmission system based on TEM horn antennas and photoconductive switches. By replacing traditional pulse voltage generators and coaxial waveguide junctions with picosecond laser pulse-triggered photoconductive switches, the system significantly reduced the rise time of electromagnetic pulses, enabling radiation of nanosecond-width pulses with picosecond-level rise times.

Many existing studies have proposed methods for designing and optimizing wideband electromagnetic pulse radiation antennas. However, current research mainly focuses on applications like nuclear hardening, target identification, and electromagnetic interference. When using existing antennas in wideband electromagnetic pulse sensor calibration experiments, several limitations arise. Firstly, the low-frequency radiation performance of antennas is insufficient, and their operating bandwidth is not comprehensive enough, particularly in the very VHF band. This makes them unsuitable for wideband electromagnetic pulse signal radiation. Secondly, antennas are often large and lack the portability needed for easy setup in practical applications.

These two limitations are interconnected and difficult to balance. Pursuing high-gain antennas for long-range signal transmission often limits bandwidth, especially in the low-frequency VHF band. Conversely, expanding bandwidth to cover the VHF band can lead to large antenna size due to structural and high-voltage resistance considerations. These unresolved contradictions in existing designs affect the antennas’ applicability in wideband electromagnetic pulse sensor calibration. This study aims to enhance the antenna’s far-field radiation capability in the VHF band and improve its portability. We have designed and optimized a radiation antenna to address these issues.

## 2. Analysis of the Pulse Radiation Characteristics of TEM Horn Antennas

**Figure 1:**
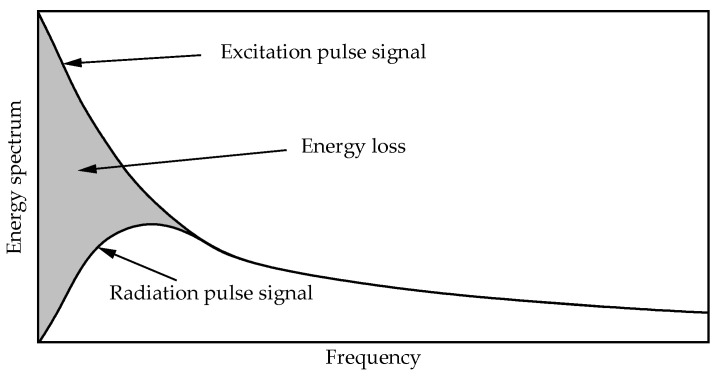
Schematic diagram comparing pulse excitation and radiated signal energy spectrum.

Due to the transient characteristics of electromagnetic pulse signals, the main difference between pulse radiating antennas and conventional antennas lies in the temporal correlation of time-domain signals. Therefore, when studying electromagnetic pulse antennas, expanding relevant signals in the time-domain proves more effective. From a time-domain perspective, the far-field radiation waveform of typical electromagnetic pulse antennas generally manifests as the differentiated form of the excitation waveform. This means the rise time of the excitation pulse mainly contributes to the far-field radiation. Figure 1 shows the energy spectrum comparison between the excitation signal and radiation signal of a TEM horn antenna. The diagram reveals that due to the antenna’s limited radiation capability in low-frequency bands, only high-frequency components of the excitation signal are effectively radiated, while other portions become energy losses that fail to achieve effective radiation. Moreover, different types of signals exhibit distinct energy loss characteristics, primarily determined by the matching degree between the antenna’s operating frequency band and the signal spectrum. Energy loss increases significantly once there is a mismatch between the antenna’s operating frequency range and the signal spectrum.

The TEM horn antenna is a typical transient antenna. Its spatial configuration is formed by gradually expanding a parallel-plate transmission line according to specific apex and included angles, creating a three-dimensional horn-shaped structure. This type of antenna exhibits strong directional radiation capability. Due to its parallel-plate feeding structure, the power capacity can be enhanced by either increasing the plate spacing or filling insulating media. Based on these characteristics, TEM horn antennas have found extensive applications in high-power electromagnetic pulse radiation systems.

Farr and Baum [24] proposed that under small flare angles, the TEM horn antenna can be treated as an open-ended transmission line. By decomposing it into an infinite series of electric and magnetic dipole elements and integrating the radiation fields of axial electric and magnetic dipoles, they derived an analytical expression for the radiation electric field along the antenna’s main axis. Zhu et al. [25] performed a detailed study on the time-domain radiation mechanism of constant impedance TEM horn antennas. They examined the spatial distribution and temporal sequence characteristics of the radiation fields. Research indicates that when a TEM horn antenna is excited by an excitation signal *V*(*t*), analytical calculations reveal that the radiation field at a distance *r* along the main axis direction is mainly made up of four basic sub-waves. Sub-wave 1: transient radiation generated when the excitation pulse enters the antenna’s starting end; sub-wave 2: transient radiation from the horn aperture; sub-wave 3: transient radiation caused by reflected current at the horn aperture termination; sub-wave 4: transient radiation produced when the reflected current returns to the feed starting end.

Finally, taking the horn aperture center as the coordinate origin and temporal zero point, the four sub-waves along the main axis and their synthesized principal-axis radiation waveform are given by the following Formulas (1)–(4), respectively:

(1) $$E_{1}(r,t)=-\frac{h}{8\pi rlf_{g}}V\left(t-\frac{r}{c}\right)$$

(2) $$E_{2}(r,t)=-\frac{h}{4\pi crf_{g}}\frac{dV(t-r/c)}{dt}$$

(3) $$E_{3}(r,t)\approx -2E_{1}(r,t)$$

(4) $$E_{4}(r,t)\approx E_{1}(r,t-2l/c)$$

(5) $$E_{\mathrm{main}}(r,t)=-\frac{h}{4\pi crf_{g}}\frac{dV(t-r/c)}{dt}+\frac{h}{8\pi rlf_{g}}\left[V\left(t-\frac{r}{c}\right)-V(t-\frac{2l+r}{c})\right]$$

In these equations, *l* represents the length of the horn plate; *h* denotes the horn height; *f*_g_ is the structural impedance factor, defined as the ratio of the horn characteristic impedance *Z*_c_ to the free-space wave impedance *Z*_0_ (i.e., *f*_g_ = *Z*_c_/*Z*_0_). From these equations, it can be observed that sub-wave 2 originates from the radiation caused by the aperture field effect of the horn antenna. Comparative analysis of radiation intensity reveals that this term constitutes the dominant component of the principal-axis radiation. Moreover, its radiation field strength is directly proportional to both the time derivative of the excitation signal and the antenna effective height, while being inversely proportional to the propagation distance (*r*) and the antenna characteristic impedance (*Z*_c_).

**Figure 2:**
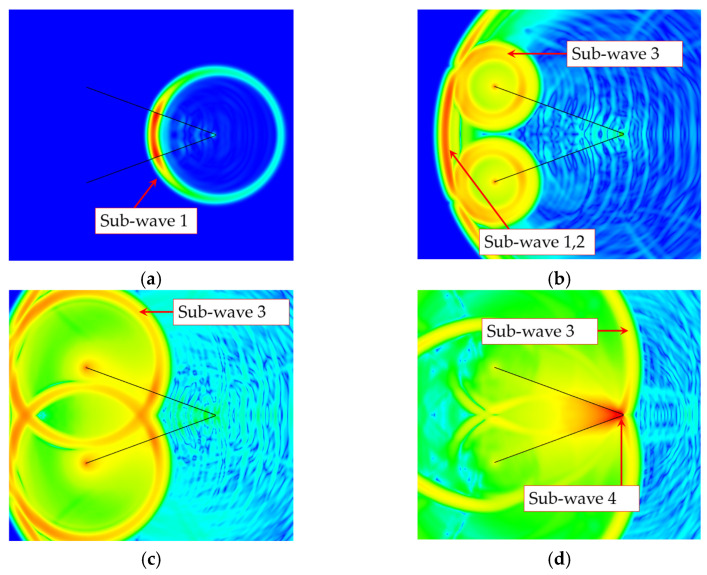
Electric field distribution around the horn antenna at different time instants. (**a**) *t* = 10 ns; (**b**) *t* = 25 ns; (**c**) *t* = 33 ns; (**d**) *t* = 38 ns.

Figure 2 illustrates the electric field distribution around the antenna at different moments, the black lines in these figures indicate the cross-sectional shape of the antenna plate. According to Formulas (1)–(4), the waveforms of sub-waves 1, 3, and 4 are proportional to the excitation voltage pulse waveform, while the radiation waveform of sub-wave 2 is proportional to the differential of the excitation voltage pulse. From the temporal sequence of sub-waves along the antenna’s main axis direction, sub-waves 1, 2, and 3 are radiated simultaneously and superimposed to form the main pulse. Sub-wave 4 is delayed by 2 *l*/c compared to the others, creating a trailing pulse. Comparing radiation intensities shows that sub-wave 2 (aperture radiation) is the main part of antenna radiation along the main axis. Sub-wave 3 follows in intensity, with an amplitude approximately twice that of sub-wave 1 but of opposite polarity, resulting in mutual weakening. Ultimately, the superposition of sub-waves 1 and 3 produces a pulse with polarity opposite to that of sub-wave 2, thereby diminishing the aperture radiation. This interference mechanism represents the primary cause of waveform distortion in the main-axis radiation of TEM horn antennas.

## 3. Antenna Design

### 3.1. High-Gain Aperture Impedance

**Figure 3:**
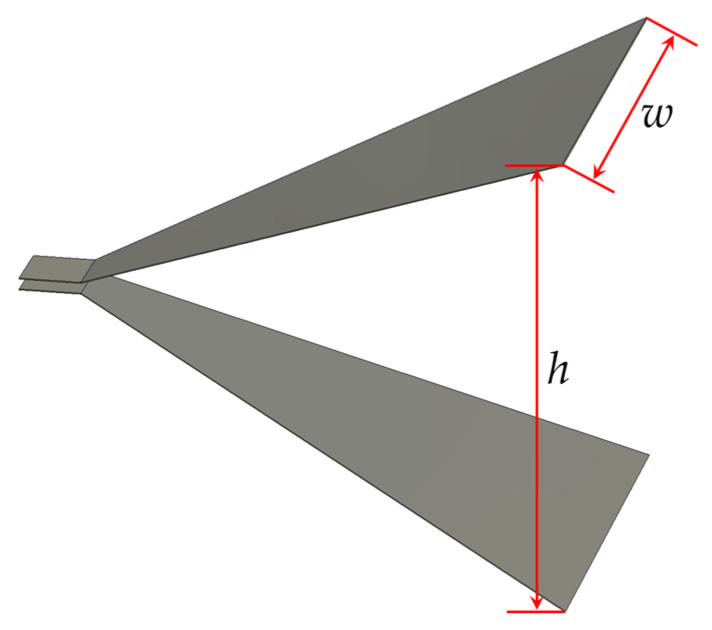
Schematic diagram of the horn antenna model.

The TEM horn antenna consists of upper and lower metal plates, as shown in Figure 3. Analogous to a parallel-plate transmission line structure, ignoring its length, the calculation method of the antenna’s characteristic impedance, shown in Formula (6), can be derived based on the transverse width (*w*) of the horn plates and the separation (*h*) between them.

(6) $$Z_{c}=\left\{\begin{array}{l} 120\ln\left(\frac{4h}{w}+\frac{w}{2h}\right)\;\;\;\;\;\;\;\;\;\;\;\;\;\;\;\;\;\;\;\;\;\;\;\;\;\;\;\;\;\;\;\;\;\;\;\;\;\;\;\;\;\;\;\;\;,\frac{h}{w}>1\\ 240\pi /\left(\frac{2w}{h}+1393+0.667\ln(\frac{2w}{h}+1.444)\right),\frac{h}{w}\leq 1 \end{array}\right.$$

Considering the impedance matching relationship between the antenna and the pulse source *Z*_R_, Equation (6) can be reformulated as

(7) $$rE(r,t)=\frac{Z_{0}}{2\pi c}\frac{dV(t)}{dt}\frac{h}{Z_{\mathrm{out}}+Z_{c}}$$

**Figure 4:**
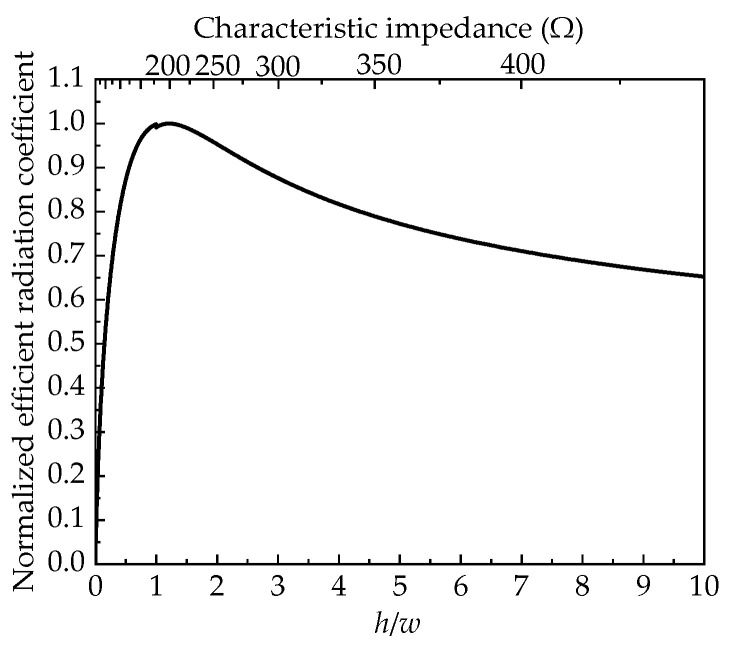
Normalized efficient radiation coefficient curve.

The efficient radiation coefficient of the antenna system can be defined as *K* = *h*/(*Z*_out_ + *Z*_c_), which is related to antenna structural parameters and intuitively demonstrates the correspondence between efficient radiation potential and antenna excitation signals. A larger efficient radiation coefficient in transient antennas indicates better far-field radiation performance and greater efficient radiation potential under equivalent excitation. Further analysis of Equation (2) shows that when the antenna aperture area and pulse source power are constant, there is an optimal impedance combination of *Z*_out_ and *Z*_c_. This combination maximizes the efficient radiation coefficient, achieving the maximum *rE*_max_. After substituting the pulse source impedance *Z*_out_ = 50 Ω, normalized analysis of the system’s efficient radiation coefficient is performed, and the results are presented in Figure 4.

**Figure 5:**
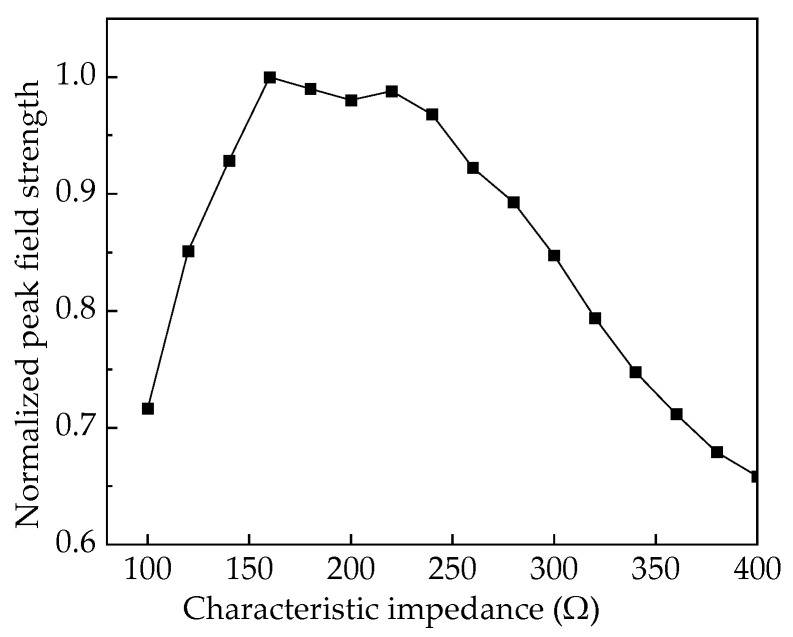
Normalized peak field strength.

The above analysis is based on ideal conditions of infinite horn length. The TEM horn antenna studied in this paper is mainly for ground testing of satellite detection payloads. Due to test site and portability limitations, the antenna’s axial length is set to 1 m, with the aperture within a 1 m diameter circumscribed circle. Modeling was performed using CST Studio Suite^®^ 2021. The excitation signal is a double-exponential pulse with a 1 kV peak voltage, 2.5 ns rise time, and 23 ns full-width at half-maximum (FWHM). The Time-Domain Finite Integration Technique (TD-FIT) was used to simulate the antenna’s radiation characteristics. Figure 5 shows the normalized peak field strengths for different antenna aperture impedances. Results show that the normalized peak field strength aligns well with the efficient radiation coefficient. As the antenna’s characteristic impedance increases, the corresponding curve first rises and then declines, peaking around 150~250 Ω.

### 3.2. Stepped Impedance Taper

After determining the high-gain aperture impedance, the TEM horn antenna aperture’s aspect ratio (height-to-width) can be established. To ensure that the impedance matches with the signal input port, TEM horn antenna design typically requires impedance matching. Adjusting the horn antenna plates’ aspect ratio can gradually vary the impedance, achieving impedance matching. Common tapering methods include linear and exponential forms. Assuming the horn’s axial length is *L*, aperture height *H*, aperture width *W*, feed-point height *H*_0_, and feed-point width *W*_0_, the height and width’s linear variations along the *z*-axis follow Equations (8) and (9).

(8) $$H(z)=\frac{H-H_{0}}{L}z+H_{0}$$

(9) $$W(z)=\frac{W-W_{0}}{L}z+W_{0}$$

**Figure 6:**
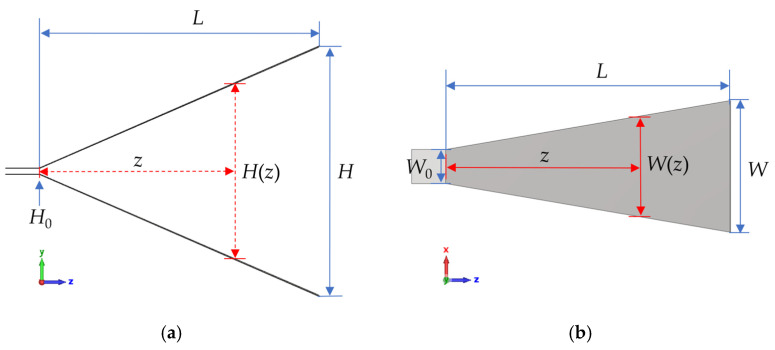
The schematic diagram of the linearly tapered TEM horn antenna. (**a**) Left view. (**b**) Top view.

The schematic diagram of the linearly tapered TEM horn antenna is shown in Figure 6.

The variation relationships of the height and width of the exponential horn along the *z*-axis direction are governed by Equations (10) and (11).

(10) $$H(z)=\frac{e^{z}-1}{e^{L}-1}\left(H-H_{0}\right)+H_{0}$$

(11) $$W(z)=\frac{e^{z}-1}{e^{L}-1}\left(W-W_{0}\right)+W_{0}$$

**Figure 7:**
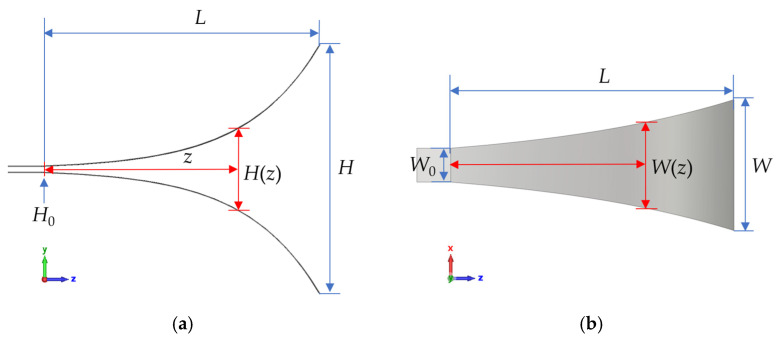
The schematic diagram of the exponentially tapered TEM horn antenna. (**a**) Left view. (**b**) Top view.

The schematic diagram of the exponentially tapered TEM horn antenna is shown in Figure 7.

**Figure 8:**
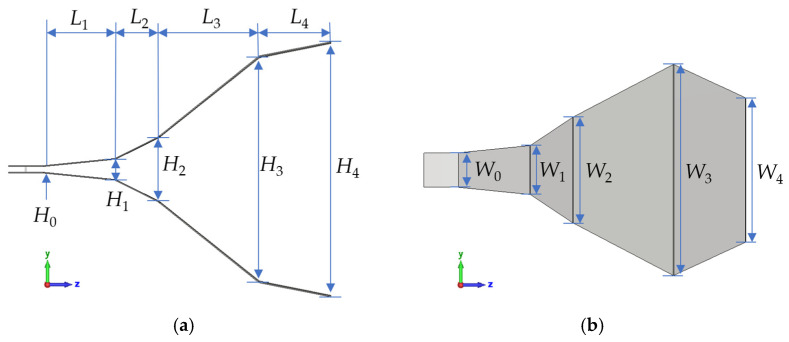
The schematic diagram of the four-section tapered TEM horn antenna. (**a**) Left view. (**b**) Top view.

Based on the height-to-width ratio determined by high-gain aperture impedance and the antenna’s size constraints, the horn antenna’s dimensional parameters are as follows: *L* = 1 m, *H* = 88.9 cm, *W* = 45.7 cm, *H*_0_ = 2 cm, *W*_0_ = 12 cm. Since conventional TEM horn antennas lack flexibility for assembly and disassembly in confined spaces, this paper proposes a four-section horn antenna structure with a four-section stepped impedance transition. The horn antenna’s schematic is in Figure 8, and each segment’s dimensional parameters are listed in Table 1.

The four-section horn antenna structure has the following characteristics:

(1) The horn’s axial length and the aperture’s circumscribed circle diameter are both 1 m.
(2) The impedance at the junctions between each pair of plates is 50 Ω, 100 Ω, 150 Ω, 200 Ω, and 250 Ω, respectively.
(3) The plate flare angles follow a “small-large-small” sequence. The characteristic impedance increases stepwise, changing slowly at the ends and rapidly in the middle.
(4) To balance high peak field strength efficiency and low-frequency radiation, the fourth-section metal plate starts at 200 Ω and ends at 250 Ω impedance.

**Figure 9:**
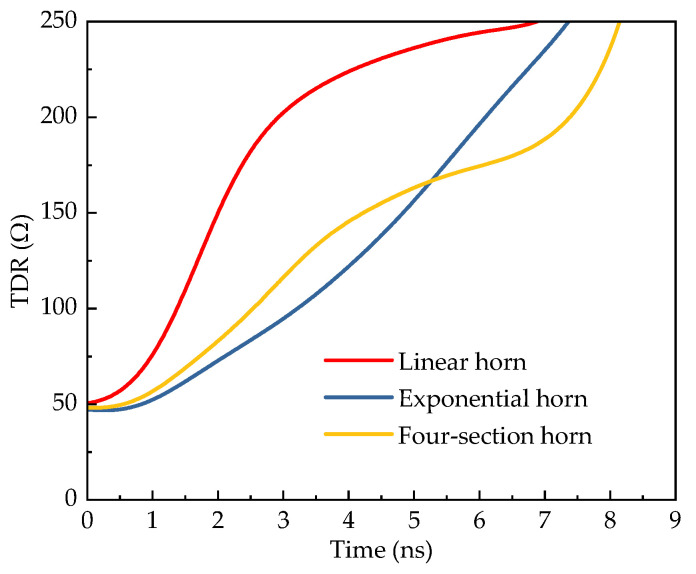
TDR simulation results of different horn antennas.

To investigate the impedance variations in three types of horn antennas, Time Domain Reflectometry (TDR) simulations were performed on each of them, with the results depicted in Figure 9. The study found that the reflective impedance of all three antennas began to rise from 50 Ω. In the initial 1 ns of signal feeding, the TDR curves showed minimal variation among the three antennas due to the feed conductor plates. However, once the excitation signal entered the tapered structure, significant differences appeared in their TDR curves. Specifically: The impedance of the linear horn increased rapidly in the early stage but slowed down later. The exponential horn displayed nearly linear impedance growth. The four-section horn showed an initial growth rate between the linear and exponential horns, followed by slow growth in the middle section and accelerated growth in the later section. Moreover, the time taken for the TDR curves of the three antennas to gradually reach 250 Ω varied. This difference was attributed to the varying plate lengths of the antennas, which resulted in a time delay corresponding to the round-trip propagation distance difference in signals on the plates.

### 3.3. End Loading of the Antenna

TEM horn antennas, due to their open-ended structure, exhibit significant terminal signal reflection, which increases the antenna reflection coefficient. As per the radiation mechanism analysis in Section 3.1, sub-wave 3 (from terminal reflections) and sub-wave 2 (from aperture field effects) have opposite polarities, adversely affecting overall radiation performance. To reduce the impact of sub-wave 3 on sub-wave 2, two methods are available: (1) Reducing sub-wave 3 intensity by using end resistance loading to interfere less with sub-wave 2. Though this approach absorbs end reflections, it also consumes some energy, reducing sub-wave 2’s intensity. (2) Modifying sub-wave 2’s polarity by installing a terminal metal plate to alter the charge acceleration direction. Then, sub-wave 2’s polarity matches sub-wave 3’s, boosting antenna radiation efficiency.

**Figure 10:**
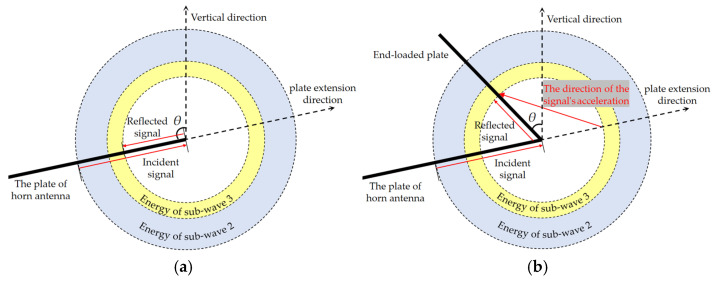
Vector analysis diagram of antenna reflection mechanism. (**a**) No load. (**b**) End-loaded metal plate.

**Figure 11:**
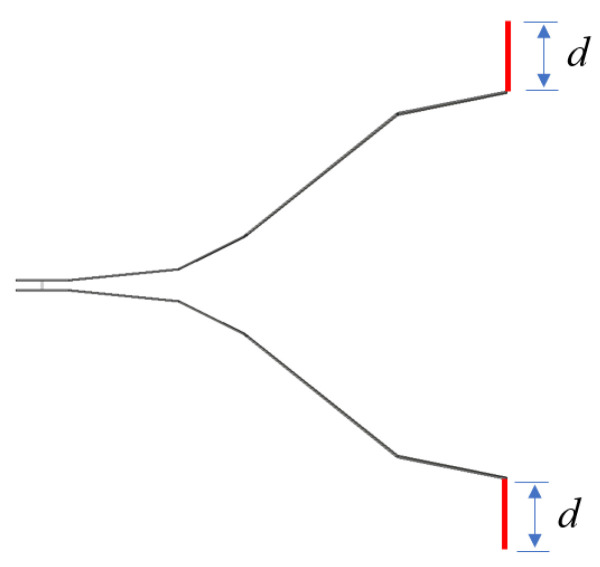
The schematic diagram of the end-loaded antenna structure.

Vector analysis of radiation mechanism at antenna terminal is based on Figure 10: Figure 10a shows the unloaded terminal configuration. When incident signals reach the plate terminal, part of the energy radiates through aperture antenna transient radiation (sub-wave 2), and the rest emits via wire antenna transient radiation (sub-wave 3). Formulas (2) and (3), respectively, represent the intensity calculation formulas of sub-waves 2 and 3 along the antenna’s main axis. These two sub-waves exhibit opposite polarities. Figure 10b presents the metallic-plate-loaded terminal configuration. When incident signals propagate to the plate terminal, sub-wave 2 retains its aperture antenna transient radiation mechanism. The current continues propagating along the loaded plate toward the opening direction. As a result, the charge acceleration orientation no longer follows the horn plate direction but shows angular dependence on *θ*. With smaller *θ* angles, sub-wave 3 achieves polarity alignment with sub-wave 2. This paper adopts the *θ* = 0° configuration to maximize the electric field strength in the main axis direction of the radiation field. The corresponding antenna loading model is presented in Figure 11. In a sense, the antenna can be regarded as a hybrid of a horn antenna and a dipole antenna with curved planes.

## 4. The Performance and Analysis of Antenna Simulation

The simulation of pulsed radiation antenna performance is a crucial step in antenna design. Numerical simulations can obtain key antenna parameters such as impedance bandwidth, input impedance, radiation efficiency, radiation pattern, and time-domain waveform. These simulation results facilitate the evaluation of the antenna’s matching performance, energy utilization efficiency, directivity, and pulse response capability.

### 4.1. Port Characteristics Analysis

#### 4.1.1. Impedance Bandwidth

**Figure 12:**
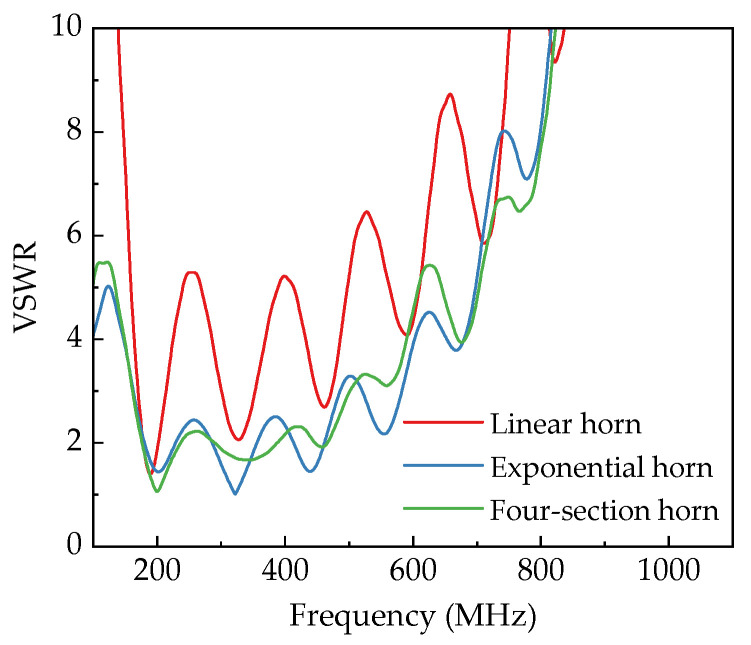
VSWR curves of different horn antennas.

**Figure 13:**
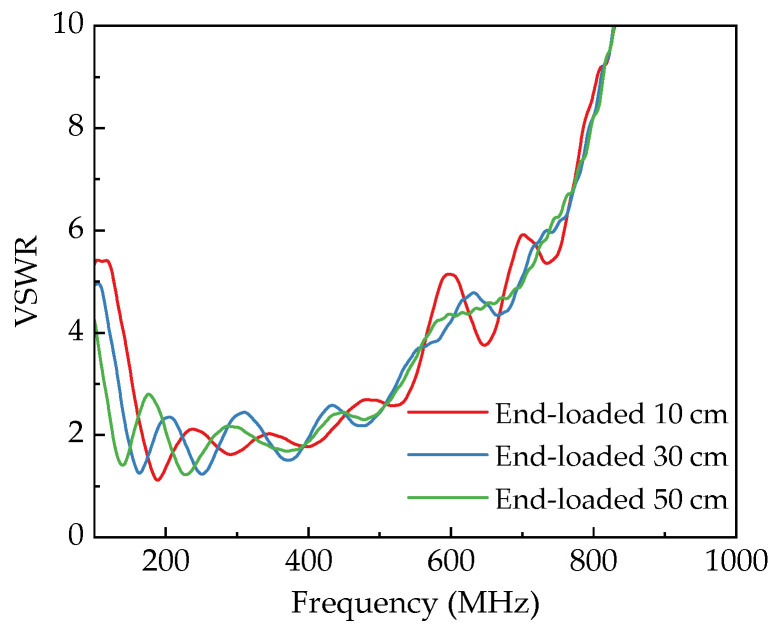
VSWR curves of four-section horn antenna with different loaded lengths.

The simulation analysis of VSWR for antennas with different taper types and loaded lengths is shown in Figure 12 and Figure 13, respectively. Comparing the VSWR curves of different taper configurations reveals the following: Under given aperture parameters and axial transition lengths, the linear horn exhibits the worst VSWR performance. Its VSWR stays below 2 only within 179~200 MHz, indicating the narrowest operational bandwidth, which fails to meet ultra-wideband requirements. Compared to the linear horn, the exponential horn shows reduced VSWR values, with significant improvement in the 200~500 MHz range. Its VSWR cutoff frequency (below 2) extends to 460 MHz at the high-frequency end, suggesting better high-frequency radiation capabilities. However, VSWR resurgence occurs in the 229~285 MHz and 356~413 MHz bands (VSWR between 2~3), indicating reduced radiation efficiency in these ranges. The four-section horn displays minimal VSWR fluctuation overall, maintaining VSWR below 3 across 161~501 MHz. Although VSWR resurgence is observed in the 154~200 MHz and 267~319 MHz bands, the amplitude of these fluctuations is notably smaller than those in the exponential horn. In summary, among the three horn antennas, the linear horn offers the poorest radiation performance; the exponential horn provides better port matching at higher frequencies; and the four-section horn delivers superior matching stability across the operational bandwidth.

A comparative analysis was conducted on four-section horns loaded with different length metal plates at their ends. The study found that loading metal plates at the antenna end shifts the VSWR curve toward lower frequencies while minimally affecting the high-frequency range, thereby improving low-frequency impedance matching. Further analysis revealed a positive correlation between the length of loaded metal plates and the degree of low-frequency extension in the VSWR curve. For instance, with the low-end cutoff frequency defined as VSWR < 3: without metal plate loading, it is 161 MHz; with 10 cm metal plate loading, it decreases to 153 MHz; with 30 cm metal plate loading, it further drops to 134 MHz; and with 50 cm metal plate loading, it lowers to 114 MHz. This confirms that end-loaded metal plates can effectively enhance low-frequency radiation performance. Simulation results show that 50 cm metal plate loading provides approximately 47 MHz of low-frequency expansion (from 161 MHz to 114 MHz), demonstrating significant performance optimization through terminal structure adjustments.

#### 4.1.2. Input Impedance Characteristics

**Figure 14:**
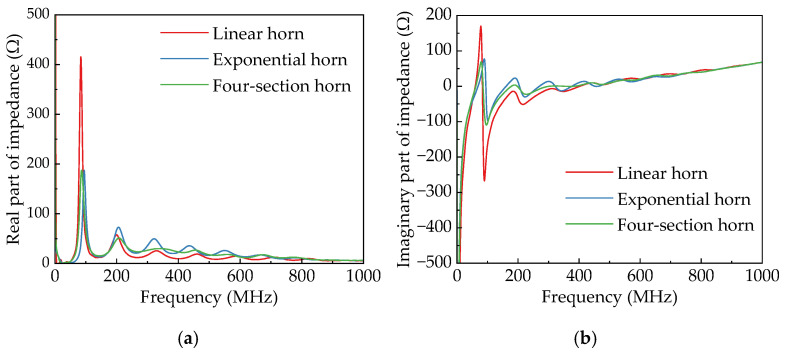
Characteristic impedance of different horn antennas. (**a**) Real part. (**b**) Imaginary part.

The input impedance characteristics of an antenna refer to the impedance presented at the input port, reflecting the antenna’s efficiency in energy exchange with the input signal. When the feed line impedance is typically 50 Ω, the antenna acts as the load for the feed line. Better impedance matching between them leads to greater real power reception and improved antenna efficiency. As shown in Figure 14, the linear horn shows the largest reactance oscillation peaks around 100 MHz, along with relatively high resistive impedance peaks. This results in poor low-frequency radiation performance. In contrast, both the exponential horn and the four-section horn have significantly reduced reactance around 100 MHz. When the frequency surpasses 300 MHz, the exponential horn and four-section horn both demonstrate better impedance matching with the 50 Ω input port. The four-section horn reaches a maximum input impedance of 187 Ω near 100 MHz, similar to the exponential horn. However, this peak occurs at a lower frequency compared to the exponential horn, indicating that the four-section horn has superior matching performance in the low-frequency range.

**Figure 15:**
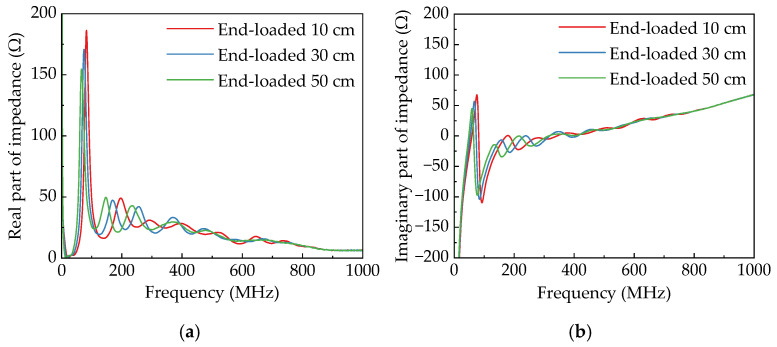
Characteristic impedance curves of four-section horn antenna with different loaded lengths. (**a**) Real part. (**b**) Imaginary part.

Comparative analysis of four-section horns with different lengths of terminal metal plates. Figure 15 illustrates impedance variations under different loading conditions. The three antennas show generally consistent trends in both real and imaginary impedance curves. When loaded with 10 cm, 30 cm, and 50 cm metal plates, the peak real impedance values are 188 Ω, 173 Ω, and 157 Ω, respectively, with maximum frequency points at 84 MHz, 76 MHz, and 68 MHz. This indicates that metal plate loading effectively improves the antenna’s port matching performance in the low-frequency band. As the length of the loaded metal plate increases, the antenna’s imaginary impedance oscillation amplitude gradually decreases, accompanied by enhanced low-frequency radiation performance.

### 4.2. Frequency-Domain Radiation Performance Analysis

#### 4.2.1. Radiation Efficiency

**Figure 16:**
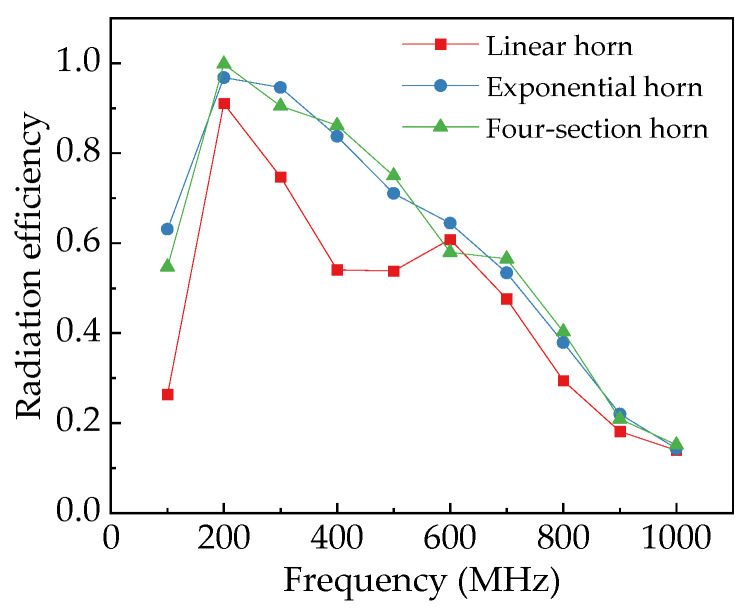
Radiation efficiency of different horn antennas.

The radiation efficiency of the antenna described here refers to its ability to effectively convert input power into radiated power at specific frequencies. This is a measure of how well electrical energy is transformed into electromagnetic wave energy. As shown in Figure 16, the radiation efficiencies of different horn antennas are relatively close at frequencies above 500 MHz. However, as the frequency decreases, the radiation efficiency of the linear horn decreases significantly. For instance, at 200 MHz, 300 MHz, and 400 MHz, the radiation efficiency values of the linear horn are 0.91, 0.75, and 0.54, respectively. In contrast, the exponential horn, due to its smoother transitional structure, demonstrates improved low-frequency radiation efficiency. At the same respective frequencies, it achieves radiation efficiency values of 0.97, 0.95, and 0.84. The four-section horn exhibits higher radiation efficiency at 200 MHz. This is primarily attributed to the minimal variation in the plate height of the *L*_4_ section (*H*_3_ = 77.4 cm, *H*_4_ = 88.9 cm) and the half-wavelength dimensions of its wire antenna configuration, which favor efficient signal radiation. At other frequencies, its radiation efficiency remains comparable to that of the exponential horn, demonstrating favorable overall performance.

**Figure 17:**
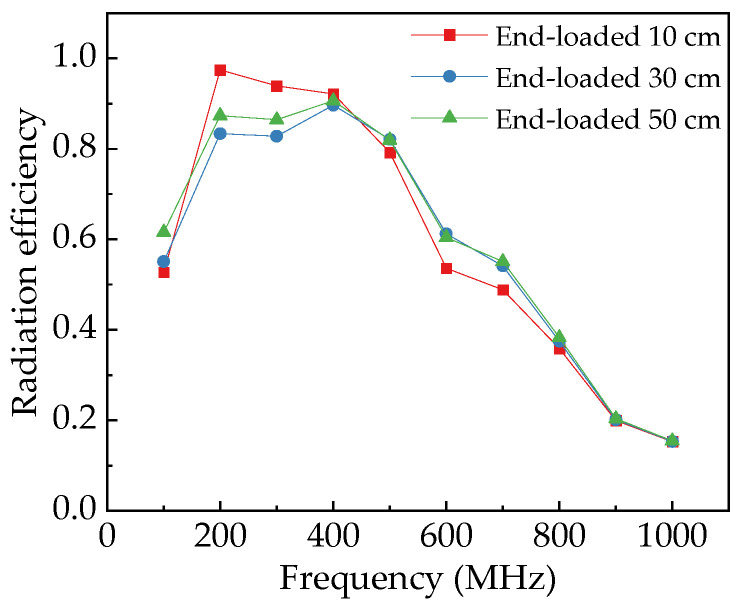
Radiation efficiency of four-section horn antenna with different loaded lengths.

The comparative analysis of four-section horn antennas loaded with metal plates of varying lengths at their terminals reveals the following findings (as shown in Figure 17): When metal plates are loaded, the radiation efficiency at 200 MHz decreases due to interference caused by the loaded plates at the aperture of the horn electrodes. Conversely, at 100 MHz, the loaded antenna demonstrates higher radiation efficiency compared to its unloaded counterpart. Moreover, as the length of the loaded metal plate increases, the radiation efficiency exhibits a progressive enhancement.

#### 4.2.2. Radiation Pattern

**Figure 18:**
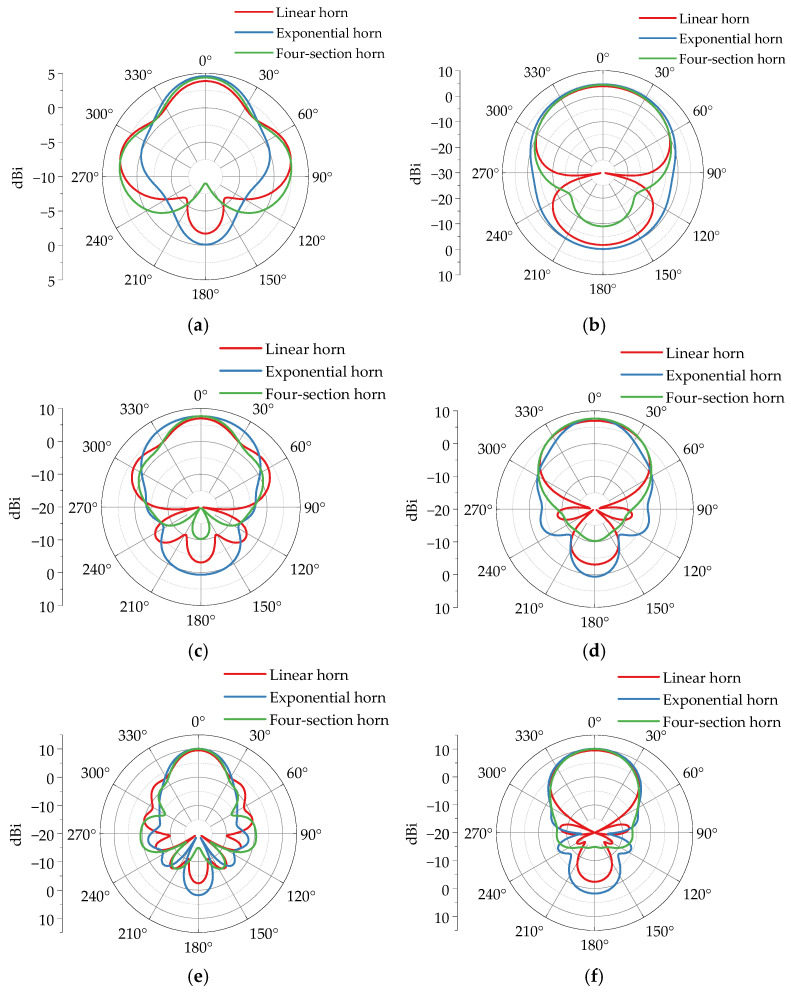
Simulation radiation patterns of different horn antennas. (**a**) *E*-plane 200 MHz. (**b**) *H*-plane 200 MHz. (**c**) *E*-plane 300 MHz. (**d**) *H*-plane 300 MHz. (**e**) *E*-plane 400 MHz. (**f**) *H*-plane 400 MHz.

The radiation pattern visually represents the radiation intensity of antennas in different directions. Figure 18 demonstrates simulation results of radiation patterns for various horn antennas. Overall, the main lobe gains of all antennas increase with frequency. Specifically, at 200 MHz, the linear horn, exponential horn, and four-section horn exhibit main lobe gains of 3.87 dBi, 4.59 dBi, and 4.38 dBi, respectively. At 300 MHz, their gains rise to 6.96 dBi, 7.63 dBi, and 7.64 dBi. At 400 MHz, gains further increase to 9.51 dBi, 10.10 dBi, and 10.00 dBi. Among the three TEM horn antennas, the linear horn displays the lowest main lobe gain and poorest radiation performance, while the exponential horn and four-section horn show comparable gains. This indicates that well-designed gradual transition structures effectively enhance antenna gain.

**Figure 19:**
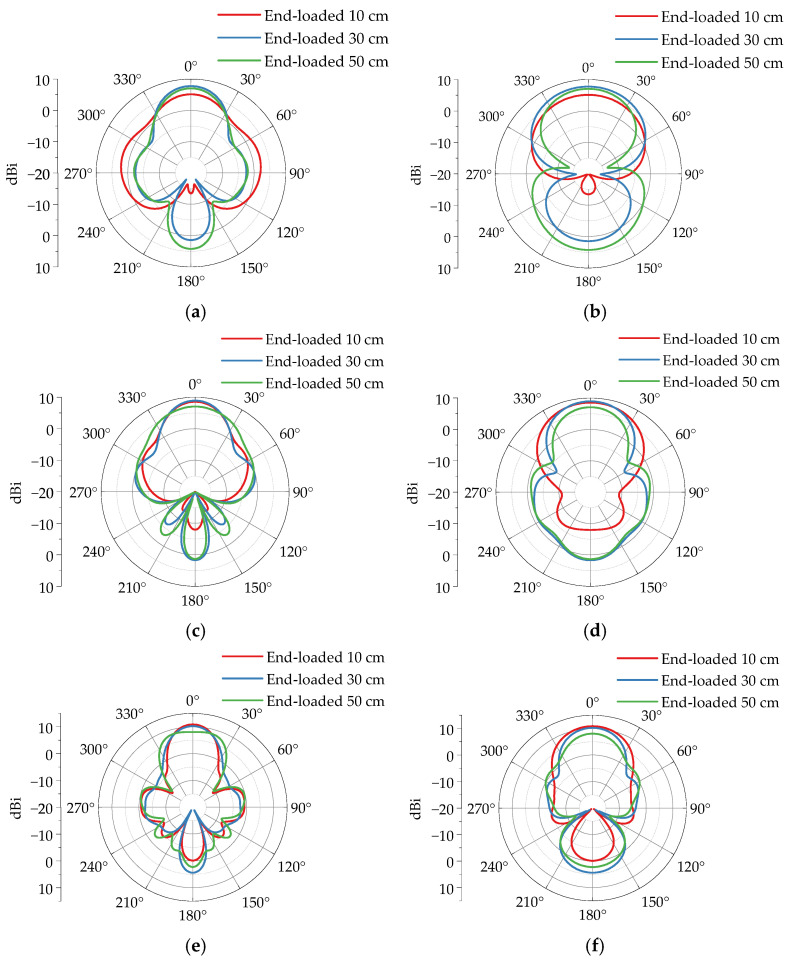
The directional patterns of a four-section horn with different loaded lengths. (**a**) *E*-plane 200 MHz. (**b**) *H*-plane 200 MHz. (**c**) *E*-plane 300 MHz. (**d**) *H*-plane 300 MHz. (**e**) *E*-plane 400 MHz. (**f**) *H*-plane 400 MHz.

Figure 19 demonstrates the far-field radiation pattern simulation results of a four-section horn antenna loaded with different length metal plates at its terminal. Regarding main lobe gain, when loaded with 10 cm, 30 cm, and 50 cm metal plates, the main lobe gains at 200 MHz are 5.12 dBi, 7.75 dBi, and 7.04 dBi, respectively; at 300 MHz, they are 8.54 dBi, 8.92 dBi, and 7.05 dBi; and at 400 MHz, the gains reach 10.90 dBi, 10.20 dBi, and 8.05 dBi. Compared to the unloaded configuration, the metal plate loading significantly enhances the main lobe gain, with the 30 cm plate achieving the maximum improvement. This indicates that as the loaded plate length increases, the antenna gain first rises and then declines after reaching a peak, with optimal radiation performance achieved using a 30 cm long metal plate.

#### 4.2.3. Current Distribution

**Figure 20:**
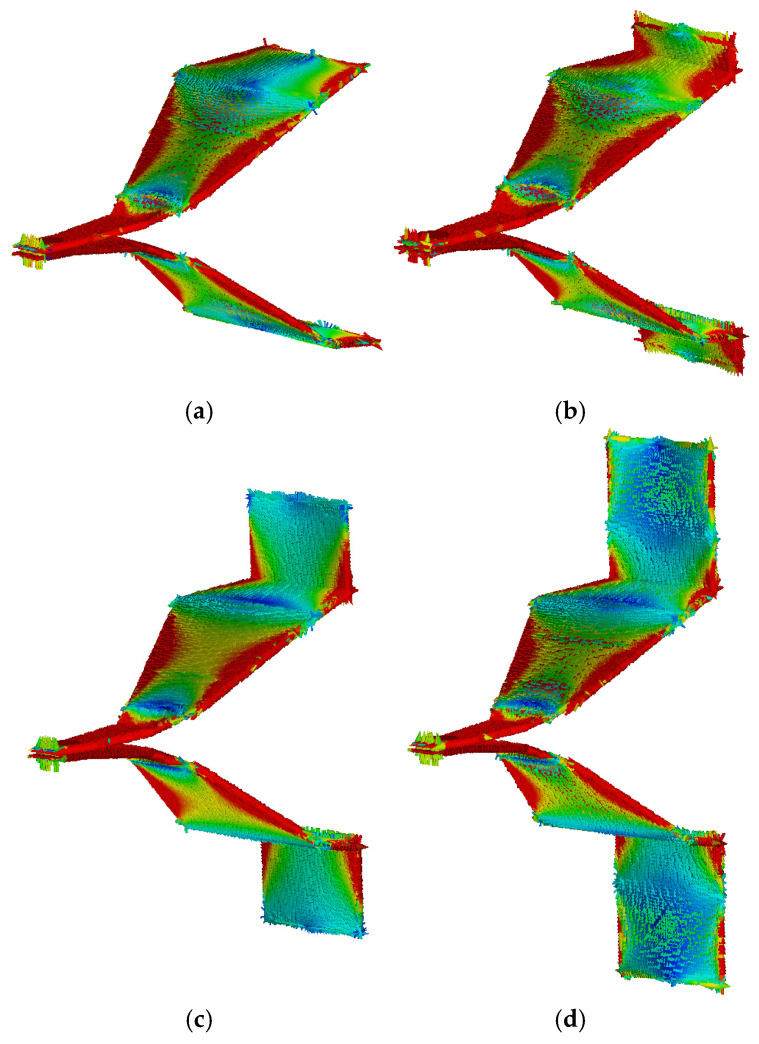
The current distribution of a four-section horn with different loaded lengths. (**a**) No-load. (**b**) 10 cm. (**c**) 30 cm. (**d**) 50 cm.

An antenna’s radiation characteristics are essentially determined by its current distribution. When the current distribution is rational and concentrated, the antenna can more efficiently radiate energy in the desired direction. Take a half-wave dipole antenna as an example. When it operates at the resonant frequency, it has a standing wave current distribution. The current is maximal in the middle and zero at the ends. This particular current distribution results in the antenna radiating most strongly in a specific direction. Therefore, this paper simulates the surface current of four-section TEM horn antennas with three different loading lengths (10 cm, 30 cm, 50 cm) under 300 MHz signal excitation. Of course, this current distribution dynamically varies, and the results presented in this paper correspond to a specific phase, as shown in Figure 20.

The surface current distribution of the four-section TEM horn antenna, as shown in Figure 20, exhibits clear standing wave effects, with the current intensity on the antenna plates alternating between strong and weak. In the original four-section TEM horn antenna shown in Figure 20a, the majority of the current zero points align precisely at the junctions of the adjacent plates. This alignment is highly advantageous for optimizing signal radiation efficiency. However, the free ends of the plates are not collocated with these current zero points, which introduces suboptimal radiation performance. This limitation motivates the implementation of end-loading design modifications to address the issue. In Figure 20b, the antenna with a 10 cm end-loaded metal plate shows a decrease in current intensity at the plate ends, but it remains high. In Figure 20c, the plate ends are close to the current zero points, so this model has better radiation performance. In Figure 20d, the current zero points are in the middle of the loaded plates, and due to the longer plate length, the overall current intensity has decreased. In summary, the antenna with a 30 cm end-loaded metal plate has the best radiation performance, which is consistent with the gain simulation results.

The physical mechanism can be explained as follows: On the one hand, when the incident signal reaches the end of the metal plate, part of the energy is reflected back. The phase of the reflected wave relative to the aperture radiation wave depends on the length of the metal plate. For the 30 cm configuration, the reflected wave undergoes a phase shift that aligns it more closely in phase with the aperture radiation wave, leading to constructive interference and enhanced radiation efficiency. On the other hand, the length of the metal plate affects the current path and distribution on the antenna. A longer metal plate allows for a more extended current path, which can influence the radiation pattern and efficiency. The 30 cm length provides an optimal balance between current path extension and phase alignment.

### 4.3. Time-Domain Radiation Performance Analysis

#### 4.3.1. Time-Domain Waveform of Pulse and Efficient Radiation Potential

**Figure 21:**
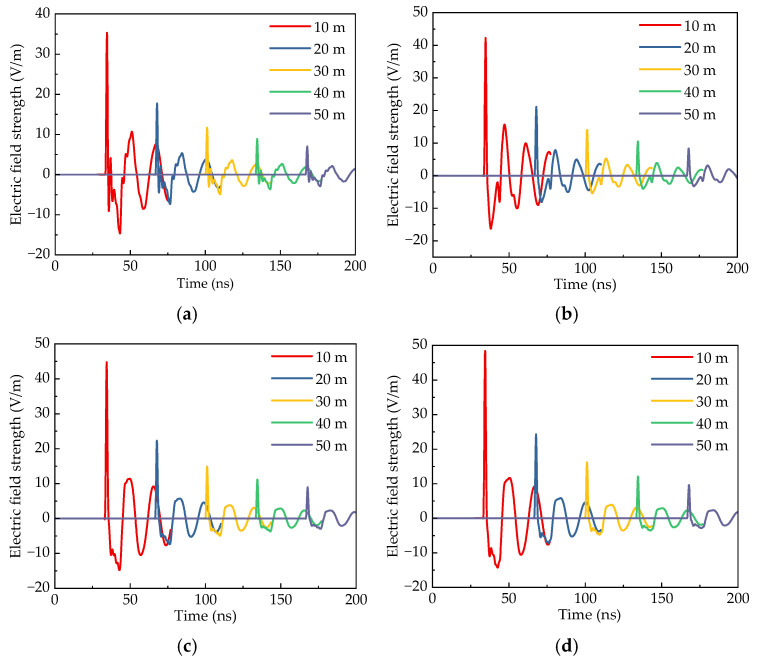

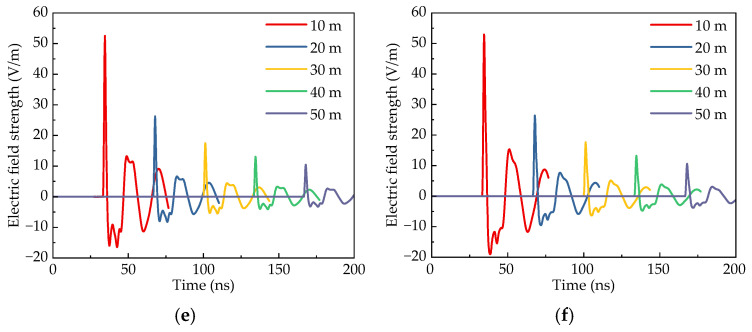
Radiation field simulation waveforms of different antennas. (**a**) Linear horn. (**b**) Exponential horn. (**c**) Four-section horn. (**d**) Four-section horn loaded with a 10 cm metal plate. (**e**) Four-section horn loaded with a 30 cm metal plate. (**f**) Four-section horn loaded with a 50 cm metal plate.

The efficient radiation potential reflects the correspondence between an antenna’s time-domain waveform and distance. By placing electric field intensity probes at 10 m, 20 m, 30 m, 40 m, and 50 m from the antenna aperture, the variation in radiation field strength with time and distance was recorded, as shown in Figure 21. The radiation waveforms at different distances show broadly similar trends, with the peak field strength gradually decreasing as distance increases.

**Figure 22:**
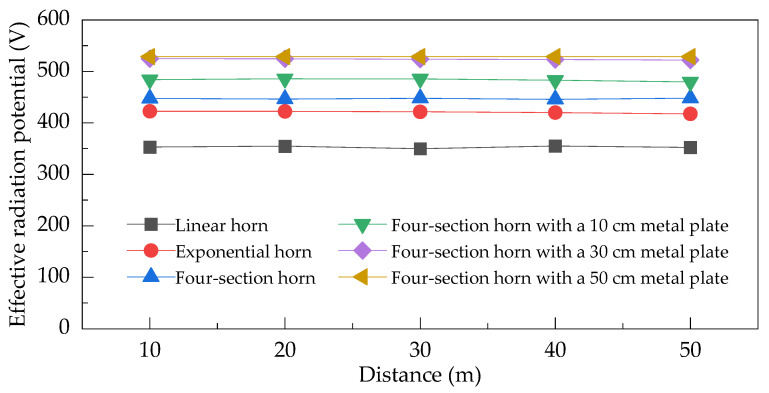
Radiation field simulation waveforms of different antennas.

To further investigate this phenomenon, a comparative analysis of the efficient radiation potentials of different horn antennas was conducted, with the results presented in Figure 22. The data in Figure 22 shows that the efficient radiation potential along the main axis direction remains relatively stable across different antenna configurations. Among the tested structures, the linear horn, exponential horn, and four-section horn exhibit efficient radiation potentials of 353 V, 421 V, and 447 V, respectively. This comparison confirms that optimized impedance transition structures significantly enhance the peak pulsed field strength. Specifically, the four-section horn achieves a 26.6% improvement over the linear configuration. The implementation of metal loading plates enhances the radiation potential through constructive interference from terminal reflections in the main radiation direction. As the loading length increases, the efficient radiation potential progressively improves, reaching 525 V at a 30 cm loading length, which is a 17.4% enhancement compared to the unloaded condition. However, beyond this threshold, further increasing the loading length yields diminishing returns, indicating saturation of the constructive enhancement mechanism. This saturation can be attributed to the temporal separation between the primary and reflected signals. When the metal loading exceeds 30 cm, the time delay (d/c) between the main pulse and terminal reflection surpasses the 1 ns pulse duration of the primary radiation. As a result, the inversely polarized reflection-induced field becomes temporally decoupled from the main pulse waveform. This temporal isolation prevents interference effects, leading to stabilized peak field strength and efficient radiation potential despite continued increases in loading length.

#### 4.3.2. Peak Field Strength Radiation Pattern

The peak field strength radiation pattern visually demonstrates the radiation intensity distribution of pulsed radiating antennas across different spatial orientations. In simulation practice, electric field probes are uniformly arranged at 10° intervals on orthogonal polarization planes centered at the antenna phase center to capture pulsed waveforms. The polar diagram generated by correlating pulse peak field amplitudes with angular deviations constitutes the antenna’s peak field strength radiation pattern. To facilitate comparison of radiation mainlobe characteristics, normalized patterns in dB scale are typically employed, where the angular span between −3 dB points defines the temporal beamwidth.

**Figure 23:**
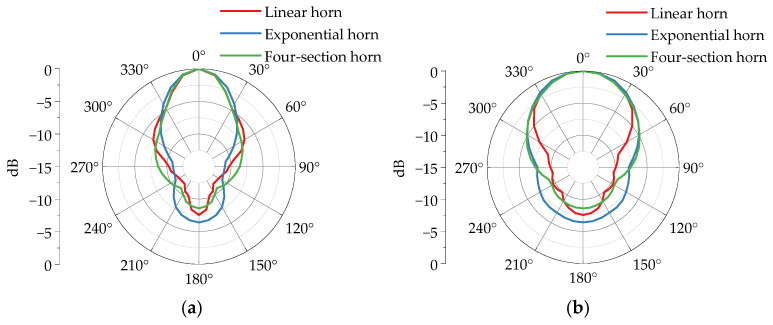
Normalized peak field strength radiation patterns of different horn antennas. (**a**) *E*-plane. (**b**) *H*-plane.

Figure 23 presents the peak field strength patterns of various horn antennas, revealing distinct mainlobe characteristics across all configurations. The *E*-plane patterns consistently exhibit narrower beamwidths compared to *H*-plane counterparts, indicating broader radiation coverage in the *H*-plane. Comparative analysis shows that exponential horns achieve the maximum *E*-plane beamwidth (49.7°), while linear and four-section tapered horns demonstrate comparable beamwidth performance. Conversely, linear horns attain the minimum *H*-plane beamwidth (78.6°), with exponential and four-section tapered designs showing similar angular coverage. Among the three tapered configurations, the four-section horn exhibits the optimal front-to-back ratio, signifying minimum backward radiation characteristics.

**Figure 24:**
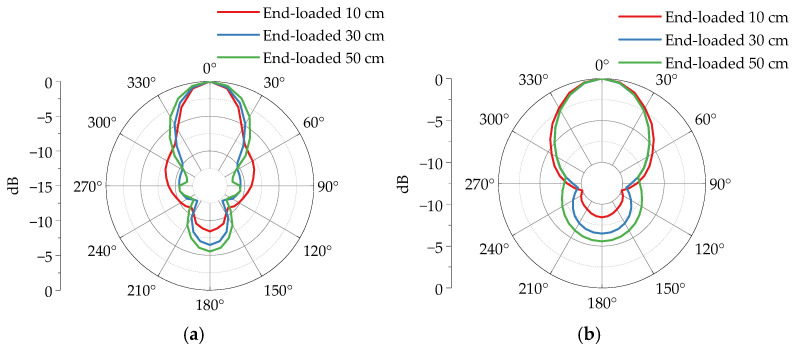
Normalized peak field strength radiation patterns of a four-section horn antenna with different loaded lengths. (**a**) *E*-plane. (**b**) *H*-plane.

Analysis of the radiation pattern characteristics of four-section antennas under different loading conditions reveals the following regularities. Comparing the simulation results in Figure 24, it is observed that loaded metal plates have a greater impact on the *E*-plane beamwidth than on the *H*-plane. As the length of the metallic plate increases, the lateral radiation energy from the horn antenna gradually decreases, while the forward/backward radiation energy progressively enhances.

According to the simulation statistics in Table 2. When the metal plate length increases from 10 cm to 30 cm, the *E*-plane beamwidth expands from 40.0° to 45.3°, the *H*-plane beamwidth reduces from 76.5° to 68.8°, and the front-to-back ratio decreases from 8.4 dB to 6.5 dB. As the plate length further increases from 30 cm to 50 cm, the *E*-plane beamwidth widens from 45.3° to 54.3°, the *H*-plane beamwidth slightly decreases from 68.8° to 68.5°, and the front-to-back ratio drops from 6.5 dB to 5.6 dB. These results demonstrate that metal plate loading effectively reduces the beamwidth disparity between *E*-and *H*-planes, balancing the energy distribution of the main lobe across both planes. However, this configuration concurrently increases backward radiation. Therefore, optimized design requires careful consideration of specific application requirements to balance these effects.

## 5. Antenna Testing

**Figure 25:**
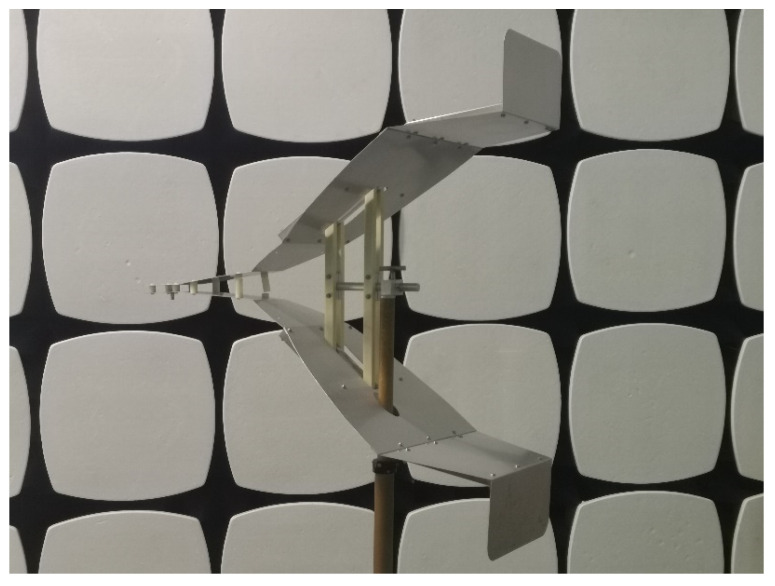
Photograph of the four-section TEM horn antenna.

To validate the antenna performance, a four-section TEM horn antenna with 30 cm end loading was fabricated and tested. The photograph is shown in Figure 25, the entire antenna adopted a modular structural design, enabling convenient disassembly and assembly. The antenna plates were made of aluminum, chosen for its excellent electrical conductivity and ease of processing and shaping. Nylon rods were used at support positions for fixation, ensuring both insulation and mechanical stability. Notably, the antenna incorporates two slots near the center of its plate for overall antenna fixation. These slots, with a narrow width of less than 5 cm, were found to have a negligible impact on the antenna’s radiation performance through pre-fabrication assessments and simulations that included all structural details. Additionally, chamfering treatment was applied to partial edges of the radiating plates to minimize the sharp-edge effect and optimize electromagnetic radiation performance.

### 5.1. Measuring Instruments and Their Operating Methods

#### 5.1.1. Wideband Electromagnetic Pulse Signal Source

**Figure 26:**
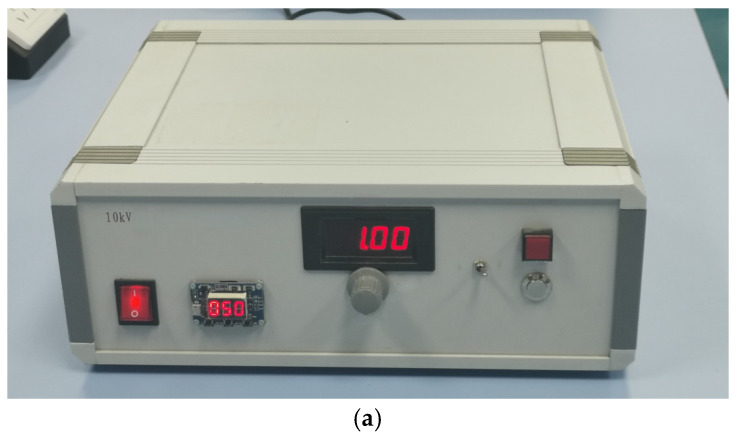

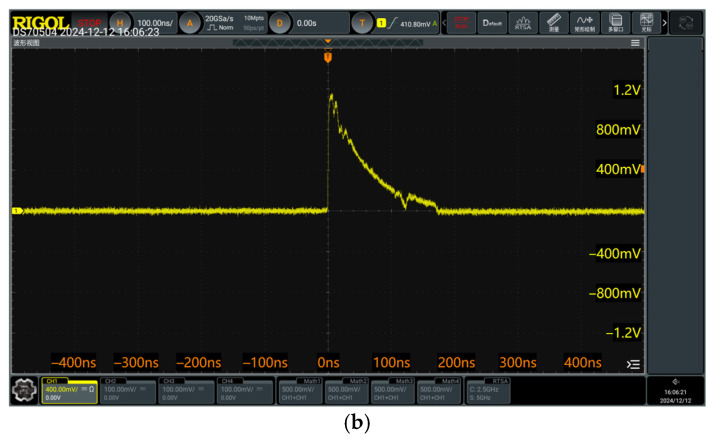
Portable wideband electromagnetic pulse signal source. (**a**) Appearance photograph. (**b**) Output waveform in the oscilloscope screenshot (after 1000× attenuation).

A self-developed 10 kV wideband electromagnetic pulse simulator is shown in Figure 26a. This device features a compact and portable design, with dimensions of 35 cm × 30 cm × 13 cm and a weight of 3 kg. The oscilloscope we used was the DS70504 model produced by RIGOL Co., Ltd., Beijing, China. When the signal source output voltage was set to 1 kV, the voltage waveform of the output signal, after passing through a 1000:1 attenuator, is illustrated in Figure 26b. The observed waveform exhibits the characteristics of a double exponential pulse signal, with a rise time of about 3.5 ns, a FWHM of approximately 42 ns, and an output voltage peak of 1.16 V. This performance meets the experimental requirements for electromagnetic pulse signal testing.

#### 5.1.2. Electric Field Sensor and Photoelectric Converter

**Figure 27:**
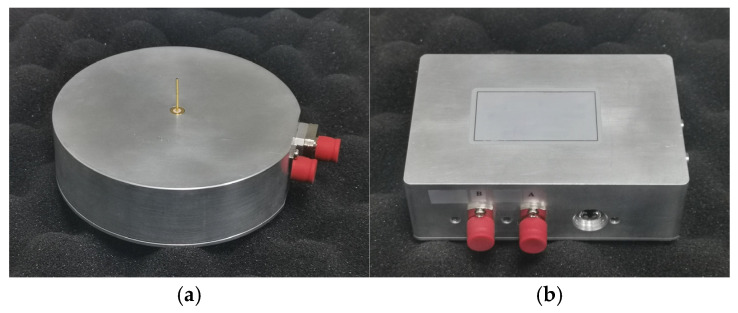
Photographs of electric field sensor and photoelectric converter. (**a**) Electric field sensor. (**b**) Photoelectric converter.

Given the characteristics of electromagnetic pulse radiation systems, such as wide signal bandwidth, fast rise time, and broad radiation range, this paper uses electric field sensors and photoelectric converters for radiation field measurement, with fiber optic transmission enhancing signal anti-interference capability. The electric field sensor, the measurement system’s core, comprises an antenna, a signal conditioning circuit, and an optical transmitter. The receiving antenna uses an electrically small monopole design. After capturing electric field signals, the antenna performs impedance transformation and signal conversion before connecting to the fiber optic link. The shielded enclosure has a disk-shaped design, ensuring excellent portability and environmental adaptability. Figure 27 shows the exterior photos of the electric field sensor and photoelectric converter.

#### 5.1.3. Sensitivity Calibration Method

**Figure 28:**
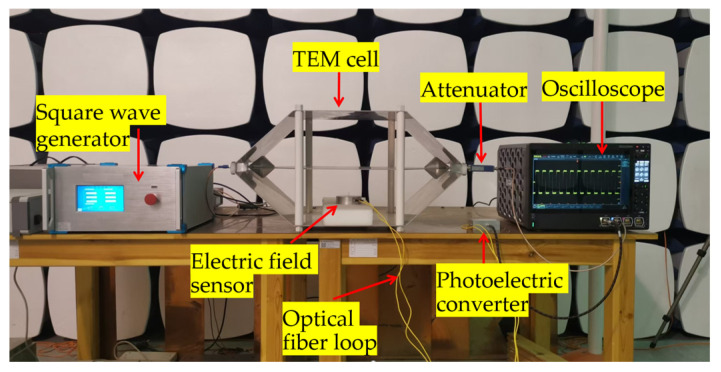
Experimental setup diagram for electric field sensor calibration.

**Figure 29:**
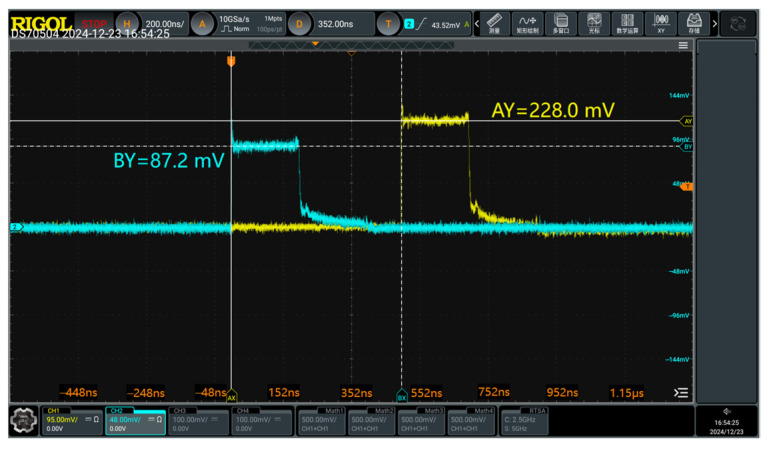
An oscilloscope screenshot during the sensitivity calibration experiment.

As shown in Figure 28, a nanosecond-level square wave generator is employed to generate standard electric fields in a TEM cell for calibrating electromagnetic field sensors. The TEM cell has a plate spacing of 20 cm, and a high-voltage attenuator with an attenuation factor of 1000 is used. During calibration, the electric field sensor is placed at the TEM cell center, and its signal is transmitted through a photoelectric conversion module to the oscilloscope’s CH1 port. The square wave generator output is connected to the TEM cell input, and the TEM cell output is linked via the attenuator to the oscilloscope’s CH2 port. Figure 29 presents an oscilloscope screenshot from the experiment. The yellow (CH1) and blue (CH2) curves show good waveform consistency, with peaks at 228.0 mV and 87.2 mV (corresponding to a reference electric field of 436 V/m in the TEM cell), respectively. By analyzing the linear relationship between the two waveforms, the sensitivity curve of the measurement system can be determined.

**Figure 30:**
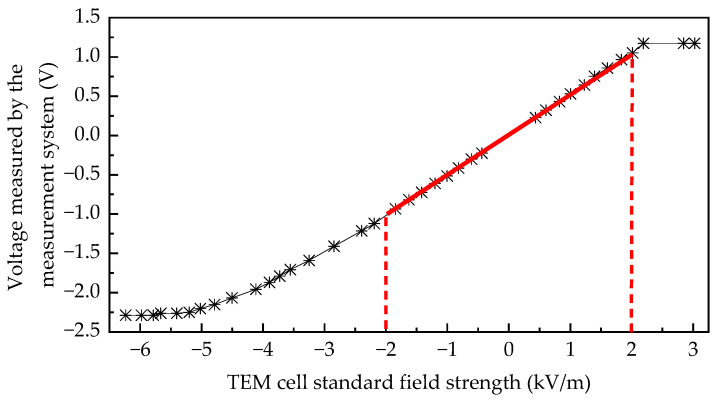
Sensitivity coefficient curve of the electric field sensor.

As shown in the sensitivity fitting curve of Figure 30, the upper measurement limit of this electric field detection system is approximately 2.2 kV/m, while the lower measurement limit is approximately −5.2 kV/m. When the electric field strength exceeds this range, measurement results may exhibit saturation phenomena. Additionally, the linearity of the sensitivity curve tends to deteriorate near the measurement limits. To ensure measurement accuracy, the effective operational range should be maintained between −2 kV/m and 2 kV/m. Within this specified range, the system exhibits a sensitivity coefficient *S*_0_ of 1.97 (V/m)/mV.

Before each measurement, the system can be calibrated using this method. The semiconductor laser in the sensor may experience thermal drift during electro-optical conversion, typically causing luminous efficiency to drop as temperature rises [26]. Factors like fiber bending/replacement and connector tightness variations can lead to optical power fluctuations, affecting the stability of the sensitivity coefficient. To address these issues, the sensor uses a standard square wave signal for calibration and employs dual-path transmission. One optical path measures the electric field signal, while the other detects the square wave signal. This allows real-time sensitivity calibration and signal continuity verification during testing. In operation, the calibration square wave amplitude *U*_1_ is measured first. The relative variation in the standard square wave amplitude (*U*_0_/*U*_1_) is proportional to optical power changes. Using this proportional relationship, the corrected system sensitivity coefficient *S*_1_ = *S*_0_ × (*U*_0_/*U*_1_) can be obtained. This approach enables comprehensive calibration of the entire measurement chain, enhancing measurement efficiency and accuracy.

### 5.2. Testing Environment and Results

**Figure 31:**
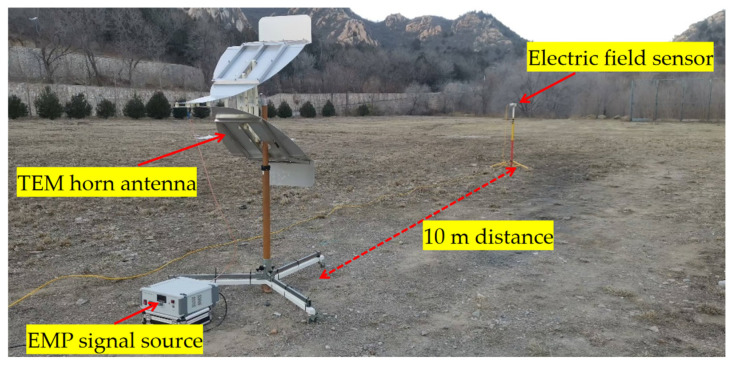
On-site layout diagram of antenna testing environment.

The antenna testing environment is shown in Figure 31. To ensure the accuracy and reliability of the test results, an open field was selected as the testing area to avoid interference from surrounding buildings and other electromagnetic sources. The electric field sensor is mounted on a nylon bracket and aligned with the horn antenna’s center at the same horizontal level to ensure measurement precision.

**Figure 32:**
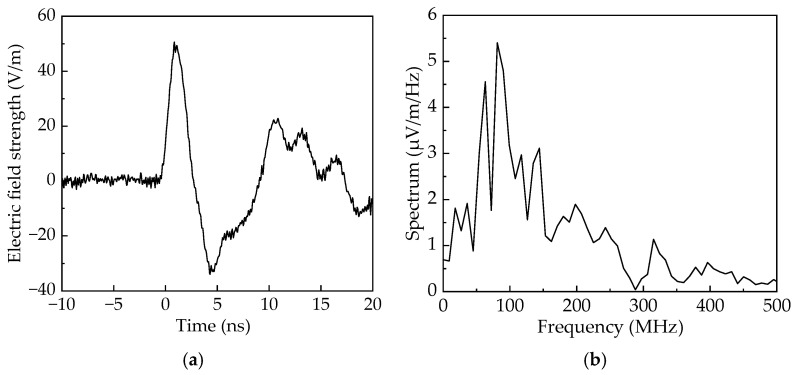
Electromagnetic pulse signal radiated by the antenna. (**a**) Time-domain waveform. (**b**) Frequency spectrum.

The measured radiation field waveform of the antenna at a distance of 10 m is shown in Figure 32a. The results demonstrate clear bipolar characteristics in the main pulse waveform, with a rise time of 0.87 ns, an FWHM of 1.75 ns, and a peak electric field strength reaching 48.9 V/m. To further analyze the spectral characteristics of the antenna, a Fast Fourier Transform (FFT) was applied to the time-domain waveform using OriginPro 2022. The resulting spectral distribution, illustrated in Figure 32b, shows a central frequency of 80 MHz and a 10 dB bandwidth ranging from 48 to 150 MHz. This corresponds to a relative bandwidth of 103%, confirming that the spectral characteristics meet the requirements for VHF ultra -wideband signals. The antenna’s performance parameters can be used for ground calibration experiments of spaceborne electromagnetic pulse sensors.

**Figure 33:**
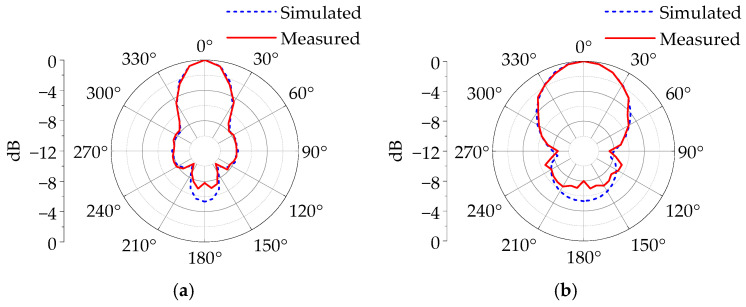
Normalized peak field strength directional diagram of electromagnetic pulse. (**a**) Time-domain waveform. (**b**) Frequency spectrum.

To investigate the pulse radiation characteristics of the antenna at different emission angles, comprehensive testing of the radiation waveforms in both the horizontal polarization plane (*E*-plane) and vertical polarization plane (*H*-plane) was performed. Transmissions were executed at 10° intervals, and a normalized radiation pattern of peak field strength was created by amplitude-normalizing the electric field intensity peaks corresponding to the antenna’s various rotational angles. The experimental results, along with simulation data, are presented in Figure 33. The results indicate that the *E*-plane and *H*-plane beamwidths are about 43° and 83°, and the sidelobe levels are approximately −8 dB and −7 dB, respectively. The measured main lobe radiation characteristics of the antenna align well with simulation, reflecting the antenna’s favorable radiation directivity. Additionally, the antenna’s cross-polarization characteristics in the main direction were measured. The results show that the cross-polarization ratio is above 22 dB, indicating good cross-polarization suppression.

## 6. Discussion

In order to underscore the originality and advantages of our proposed design, Table 3 presents a comparative summary of the measured results from our experiments and those from other electromagnetic pulse radiators. In references [19,21,22], the antennas’ radiated signals have rise times and FWHM that have not reached the nanosecond scale, implying a high signal frequency that cannot meet the low-frequency requirements for VHF ultra-wideband electromagnetic pulses. The antennas in references [18,19] do satisfy ultra-wideband electromagnetic pulse requirements in terms of pulse radiation and bandwidth. Nevertheless, their large size renders them non-portable and difficult to install and remove, making them unsuitable for ultra-wideband electromagnetic pulse detector calibration experiments. In contrast, the segmented TEM horn antenna designed in this paper not only meets the radiation signal parameter requirements for VHF ultra-wideband electromagnetic pulses but is also compact, with plates that can be easily assembled and disassembled. It has low testing environment requirements and strong environmental adaptability, making it ideal for electromagnetic pulse sensor calibration experiments.

## 7. Conclusions

This paper presents the design of an ultra-wideband four-section TEM horn antenna. It consists of three main phases. First, the aperture impedance, tapering profile, and end loading are optimized to boost far-field radiation. Next, TD-FIT analysis explores port characteristics, radiation efficiency, patterns, and potential, focusing on tapering and loading effects. Finally, a prototype is made and tested. Key findings are as follows:

(1) Optimal far-field radiation characteristics emerge when the aperture impedance is maintained between 150~250 Ω. The four-section configuration shows superior performance in voltage standing wave ratio, input impedance stability, and low-frequency radiation efficiency compared to conventional linear horns. Notably, it achieves a 26.6% enhancement in peak radiation field intensity.
(2) Vector analysis of terminal reflections informs the optimization of end loading strategies, effectively improving low-frequency radiation efficiency and peak field strength. The optimal load length correlates with the radiation pulse width, with 30 cm loading producing a 17.4% improvement in peak field, reaching 52.5 V/m compared to unloaded conditions.
(3) Implementation of a fiber-optic based self-calibrating electric field measurement system enhances the accuracy of pulsed field characterization. Measurements reveal an efficient radiation potential of 489 V, a FWHM of 1.75 ns, a center frequency of 80 MHz, and a 10 dB bandwidth of 48~150 MHz. Radiation patterns show 43° *E*-plane and 83° *H*-plane beamwidths. The developed system is practically portable for electromagnetic pulse simulation applications and is viable for ground calibration testing of spaceborne wideband electromagnetic pulse sensors.

## Figures and Tables

**Table 1:** Dimensions parameters of the four-section tapered TEM horn antenna.

X	0	1	2	3	4
*L*_x_ (cm)	12.0	25.0	15.0	35.0	25.0
*W*_x_ (cm)	12.0	17.2	37.0	63.3	45.7
*H*_x_ (cm)	2.0	7.0	22.0	77.4	88.9
*Z*_c_ (Ω)	50	100	150	200	250

**Table 2:** Statistical table of simulation data for normalized peak field strength radiation patterns.

	*E*-Plane Beamwidth	*H*-Plane Beamwidth	Front-to-Back Ratio
Linear horn	41.9°	78.6°	7.6 dB
Exponential horn	49.7°	85.9°	6.4 dB
Four-section horn	41.6°	83.7°	8.6 dB
Four-section hornend-loaded 10 cm	40.0°	76.5°	8.4 dB
Four-section horn end-loaded 30 cm	45.3°	68.8°	6.5 dB
Four-section horn end-loaded 50 cm	54.3°	68.5°	5.6 dB

**Table 3:** Comparison of the performance among different radiation equipment.

Ref.	Type of Antenna	Approximate Size	Radiated Impulse Signal		
			Rise Time	Pulse Width	Bandwidth
[18]	Reflector antenna with TEM horn feed	20 m × 20 m × 25 m	1.9 ns	2.5 ns	8~205 MHz
[19]	4-element TEM horn array	0.24 m × 0.24 m × 0.3 m	30 ps	~1 ns	/
[20]	TEM horn antenna	3 m × 3 m × 3 m	2.5 ns	20.9 ns	<300 MHz
[21]	16-element TEM horn array	1 m × 1 m × 2 m	0.12 ns	0.18 ns	/
[22]	4-element TEM horn array	0.4 m × 0.4 m × 0.6 m	60 ps	80 ps	0.1~6 GHz
[23]	TEM horn antenna	0.3 m × 0.3 m × 0.3 m	<30 ps	~1 ns	/
This paper	Segmented TEM horn antenna	1 m × 1 m × 1 m	0.87 ns	1.75 ns	48~150 MHz

## Data Availability

Data are contained within the article.
